# Novel nanobody-161 binds tumor necrosis factor receptor 2 (TNFR2) to exert an anti-tumor effect but does not block TNFα-binding

**DOI:** 10.3389/fimmu.2025.1694313

**Published:** 2025-12-08

**Authors:** Liang Huang, Chao Kong, Yuhao Hu, Michael Zhang, Shuangqi Li, Wanjing Wang, Hui Guan, Mei Ling Lim, Zhen Zhang, Wenhai Zhang, Hailiang Zheng, Xintian Yan, Xinglu Sun, Pan Lei, Shanshan Dai, Wenrong Wang, Linlin Lu, Junma Zhou, Shilong Fan, Guojun Lang

**Affiliations:** 1 Sanyou Biopharmaceuticals Co., Ltd., Shanghai, China; 2 Shanghai Institute of Immunology, Department of Immunology and Microbiology, Shanghai Jiao Tong University School of Medicine (SJTU-SM), Shanghai, China; 3 Spesbio (Beijing) Co. Ltd., Beijing, China; 4 Beijing Frontier Research Center for Biological Structure, Tsinghua University, Beijing, China

**Keywords:** tumor necrosis factor receptor 2, nanobody-161, TNFα, T cells, antibody-dependent cell-mediated cytotoxicity

## Abstract

**Background:**

Tumor necrosis factor receptor 2 (TNFR2) is expressed on regulatory T cells (Tregs) and many cancer cells indicating its potential as a therapeutic target. Traditional non-blocking antagonists often disrupt immune homeostasis by excessively suppressing Treg cell function when blocking the TNFα signaling pathway, weakening the body’s antitumor immune response. A novel anti-cancer mechanism for targeting TNFR2 is presented and clinical potential discussed.

**Methods:**

A novel humanized anti-TNFR2 nanobody, Nanobody-161, was identified from the Sanyou Bio Super Trillion Antibody Library in the current work and the non-blocking effect of Nanobody-161 in TNFα-TNFR2 signaling investigated. A transgenic mouse model was established to investigate its anti-tumor activity. The crystal structure of the complex with TNFR2 was also analyzed.

**Results:**

Nanobody-161 had antitumor activity in a transgenic mouse model, reducing tumor weight by 5-fold at a dose of 7.5 mg/kg and inhibited TNFα-TNFR2 signaling in HEK293 cells overexpressing human TNFR2 with 10-fold greater potency than traditional antagonists. Nanobody-161 was not observed to disrupt TNFα-induced Treg proliferation in peripheral blood mononuclear cells. Nanobody-161 mediated Fc-dependent CD8+ T cell activation and TNFR2+ Tregs in the tumor microenvironment were depleted by antibody-dependent cell-mediated cytotoxicity (ADCC). The structure of Nanobody-161 VHH in complex with TNFR2 was determined at 2.9 Å resolution and epitopes of TNFR2 CRD2 and CRD3 were identified. Nanobody-161 may inhibit TNFR2 oligomerization but was not observed to block TNFα binding.

**Conclusion:**

Nanobody-161 is a novel non-blocking TNFR2-antogonist that inhibits tumor growth without causing immunosuppression and is a promising candidate for safer and more effective therapy of solid tumors.

## 1 Introduction

Antibody-based therapies target tumor-associated antigens (TAAs) or tumor-specific antigens (TSAs), stimulating antibody-dependent cell-mediated cytotoxicity (ADCC) and causing complement-dependent cytotoxicity (CDC) or apoptosis (1, 2). Tumor growth, invasion and metastasis may also be reduced by blockade of signal transduction (3, 4). However, tumors may downregulate tumor antigen expression, major histocompatibility complex (MHC) proteins or upregulate the secretion of immunoinhibitory cytokines, suppressing the anti-tumor response of cytotoxic T lymphocytes (CTL) and natural killer (NK) cells (5, 6). Such mechanisms allow the tumor to evade immune surveillance. Therefore, new therapies are needed to block the inhibitory receptors expressed on the immune cell surface, enabling the reactivation of the immune system.

Immune checkpoint blockade (ICB) therapies against programmed death protein 1 (PD-1), programmed cell death ligand 1 (PD-L1), lymphocyte-activation gene 3 (LAG-3) and CTL antigen 4 (CTLA-4) are FDA-approved for over 50 types of cancer, including melanoma, non-small cell lung cancer, small cell lung cancer, renal cell cancer, head and neck squamous cell carcinoma, colorectal cancer and hepatocellular carcinoma (7, 8). Pembrolizumab and nivolumab are first‐line therapies for advanced cutaneous melanoma which produce overall response rates of 30% to 40%, increasing to about 60% in combination with ipilimumab (9). The principal action of ICB therapy is to restore T cell activity and therapeutic efficacy varies with tumor type, meaning that many patients do not benefit from immunotherapy and may experience immune-related adverse reactions that compromise quality of life. Efficacy is limited in acral melanoma, triple-negative breast cancer and pancreatic cancer, for which combination therapies are recommended (10, 11). These observations illustrate the potential of combinations of antibodies to enhance treatment and reduce toxicity. Therefore, developing novel immunotherapy strategies, particularly those targeting specific immunosuppressive mechanisms within the tumor microenvironment, is of paramount importance.

Tumor necrosis factor (TNF) binds a cognate receptor, TNF receptor 1 (TNFR1) (or TNFRSF1B), to promote apoptosis and an alternative receptor, TNFR2, to exert an opposing pro-survival effect (12, 13). TNFR2 shows limited expression in the tumor microenvironment (TME) but is highly expressed on regulatory T cells (Tregs) and myeloid-derived suppressor cells (MDSCs) (14). It is also abundantly expressed on many tumor cells, including those involved in ovarian (15), esophageal (16), breast (17) and colon (18) cancers. Since TNFR2 is not widely expressed in the TME, it is a suitable therapeutic target for agonists and antagonists aimed at manipulating the fate of Tregs (19). Antibodies raised against the TNFα binding site of TNFR2 inhibited the proliferation of TNFR2+ Treg cells at low dosage and eliminated Tregs from ovarian cancer ascites and TNFR2+ OVCAR3 cells (15). TNFR2 antagonist monotherapy had anti-tumor effects *in vivo* and exerted a synergistic immune response when combined with ICB therapies (20–23). Several similar anti-TNFR2 antibodies, including OPI SBT002e, BI-1808 and SIM-1811-03, have undergone clinical trials or preclinical testing (24–26) and OPI-SBT002e was selected as the representative antibody in the current study. Anti-TNFR2 antibodies with TNFα agonist activity promote TNFR2+ Treg cell proliferation and have been used to treat autoimmune diseases (27). LBL-019 blocked the TNFR2-TNFα interaction whereas HFB200301 was not observed to affect TNFα binding (28, 29). Both these agonists stimulated CD4+ and CD8+ T cells and had antitumor activity either alone or in combination with anti-PD-1 (30).

The anti-TNFR2 antibodies listed above are predominately native full-length IgGs. Nanobodies are shorter versions, consisting of a single heavy chain variable region (VHH) and incorporating the constant domains, CH2 and CH3, but lacking light chains. Nanobodies have dimensions of 2.5 nm diameter and 4 nm length and constitute the smallest antibody fragment which retains antigen-binding capacity (31, 32). Nanobodies are suitable candidates for design of immunotherapeutic drugs due to their low molecular weight, high tumor tissue penetration, stability and the ease with which bispecific antibodies may be generated (33–35).

Nanobody-161 is introduced as a novel non-blocking anti-TNFR2 nanobody and its anti-tumor activity investigated in a C57BL/6_hTNFR2 transgenic mouse model in the current work. The crystal structure was solved and domains responsible for receptor binding were investigated with a view to identifying the nature of its non-blocking action. The crystal structure is the first anti-TNFR2 nanobody to be entered into the protein data bank (PDB). The aim of the study was to evaluate the potential of Nanobody-161 as a novel therapeutic strategy for human cancer.

## 2 Materials and methods

### 2.1 Recombinant protein

#### 2.1.1 Ligand proteins

TNFα-His (trimer) was purchased from ACROBiosystems (TNA-H5228). For TNFα-Fc dimer construction, the C-terminal region of the human TNFα gene obtained from the NCBI database (accession no.: NP_000585.2) was linked to the human IgG1 Fc segment and the construct was inserted into the eukaryotic expression vector, pcDNA3.4-TOPO (Invitrogen), and expressed using the ExpiCHO transient expression system (Gibco, A29133). The supernatant was filtered through a 0.22 μm filter and purified using Protein A/G affinity purification and 100 mM glycine salt (pH 3.0) (36). For TNFα-His construction, the C-terminal region of the human TNFα gene was labeled with a hexahistidine tag, inserted into the eukaryotic expression vector, pcDNA3.4-TOPO (Invitrogen), expressed using the ExpiCHO transient expression system (Gibco, A29133), the conditioned medium filtered through a 0.22 μm filter, purified by nickel immobilized metal affinity column (IMAC) (37) and eluted with 500 mM imidazole.

#### 2.1.2 TNFR2 antigen proteins

Human, monkey and mouse TNFR2 recombinant proteins were purchased from Sino Biological (10417-H03H, 90102-C08H and 50128-M08H).

### 2.2 Screening and generation of anti-TNFR2 nanobody

Nanobody-161 was screened from the Super Trillion Antibody Library (STAL, Sanyou Bio) using liquid phase magnetic beads linked to recombinant TNFR2 protein, optimized by humanization and affinity maturation. Nanobody-161 IgG1 was prepared using the ExpiCHO system (Thermo Fisher Scientific). Briefly, plasmids containing sequences encoding the heavy-chain and light-chain were mixed in ExpiFectamineTM CHO, added to the cell suspension and mixed gently. The cell culture was incubated at 37°C and 7% CO_2_ with shaking. ExpiCHOTM Enhancer and ExpiCHOTM Feed were added 18–22 h after transfection and the cell culture incubated at 32 °C with 5% CO_2_. The conditioned medium containing the antibody was collected 7–15 days after transfection, centrifuged at 12,000 × g for 10 min, the supernatant affinity purified with MabSelect SuRe LX (GE) and antibody eluted with 100 mM Glycine-HCl (pH 3.0) and neutralized with 1 M Tris-HCl. A centrifugal filter unit (Millipore) was used to exchange the purified antibody into PBS buffer and aliquots were stored at - 80 °C. OPI-SEB002e, Y9, SIM-0235–001 and BI-1808 were also expressed by the ExpiCHO transient system and purified as above. Bacterial endotoxin levels were determined using the Chromogenic LAL Endotoxin Assay Kit (Beyotime) and endotoxin levels were <1 EU/mg before any *in vitro* and *in vivo* antibody testing.

### 2.3 Generation of huTNFR2-HEK293 and huTNFR2-Jurkat cells

The full-length human TNFR2 DNA sequence was synthesized via Sangon Biotech company, cloned into an expression vector, pLVX-puro, transfected into Escherichia coli DH5α with selection by puromycin resistance and the plasmid extracted and sequenced. HEK293 cells (ATCC^®^ CRL-1573TM) were cultured in DMEM (Gibco, 11995-665) and Jurkat cells (ATCC^®^ TIB-152) in RPMI1640 (Gibco, 11875093). Cells were subcultured to 5 × 10^5^ cells/mL and plasmids introduced by electroporation kit (Invitrogen, MPK10096) and electroporation apparatus (Invitrogen, NeonTM Transfection System, MP922947) the following day. Cells were cultured in DMEM containing 10% FBS (Thermo Fisher, 10099141) at 37 °C for 48 h. Cells were plated into 96-well plates at a density of 1500–4000 cells/well, 2 μg/mL puromycin (Gibco, A1113803) added for 10 days and surviving clones transferred to 24-well plates (Corning, 3599). Cell lines expressing human TNFR2 were identified using antibody SBT002e by flow cytometry (Beckman Coulter, CytoFLEX AOO-1-1102). Jeko-1 cells derived from peripheral blood mononuclear cells of a patient with a large cell variant of mantle cell lymphoma (MCL) were purchased from Institute of Basic Medical Sciences, Chinese Academy of Medical Sciences.

### 2.4 Enzyme linked immunosorbent assay (ELISA) detection of antibody binding to recombinant proteins

ELISA plates (Greiner, 655209) were coated with 2 μg/mL human/monkey/mouse TNFR2 recombinant protein and incubated overnight at 4 °C. Plates were washed three times with phosphate buffered saline with tween (PBST), blocked with 5% skimmed milk for 2 h at room temperature, washed three times with PBST and antibodies added for 1 h. Plates were washed three times with PBST, NeutrAvidin-HRP (1:4000, Thermo Fisher, 31001) added for 1 h at room temperature, plates washed six times with PBST and 3,3’,5,5’-Tetramethylbenzidine (TMB) chromogen (SurModics, TMBS-1000-01) added for color development in the dark for 5–10 min. 2 M HCl was added to stop the reaction and OD450 read by microplate reader (Molecular Devices, SpectraMax 190).

### 2.5 Flow cytometry detection of antibody binding

Cultured huTNFR2-HEK293 and huTNFR2-Jurkat cells were collected, centrifuged at 300 g, cell pellets resuspended in Fluorescence-activated cell sorting (FACS) buffer (PBS containing 2% FBS), counted and 100 μL/well of a 1 × 10^6^ cells/mL suspension plated into 96-well round bottom plates. Serially diluted antibodies were added with incubation at 4 °C for 30 min., cells were washed three times with FACS buffer and PE-labeled anti-human IgG Fc flow antibody (1:5000, Abcam, ab98596) added. Cells were resuspended and incubated at 4 °C for 30 min., washed three times with FACS buffer, resuspended and antibody signals were detected by flow cytometry (Beckman Coulter, CytoFLEX AOO-1-1102).

### 2.6 Flow cytometry detection of antibody blocking activity

Cultured huTNFR2-HEK293 cells were collected, centrifuged at 300 g, resuspended in FACS buffer, counted and 100 μL/well of a 2 × 10^6^ cells/mL suspension plated into 96-well round bottom plates. Serially diluted antibodies were added with incubation at 4 °C for 30 min, cells washed three times with FACS buffer and 0.1 μg/mL biotin-labeled TNFα-Fc fusion protein dimer added with incubation at 4 °C for 30 min. Plates were washed three times with FACS buffer, PE-labeled streptavidin (1:5000, eBioscience, 12-4317-87) added, plates incubated at 4 °C for 30 min., washed three times with FACS buffer and 200 μL FACS buffer added per well for detection of binding by flow cytometry (Beckman Coulter, CytoFLEX AOO-1-1102).

### 2.7 Protein expression and purification for crystallization

#### 2.7.1 TNFR2 antigen production

The gene encoding the TNFR2 ectodomains (residues 33-205; Uniprot ID: P20333) was cloned into the pET-22b vector and expressed in Escherichia coli BL21(DE3) as inclusion bodies. TNFR2 was renatured by dialysis and purified as follows: cells were grown in LB medium at 37 °C until an OD 600 of 1.0 was reached. Protein expression was induced with 1 mM IPTG, cells cultured for 4 h at 37°C, collected by centrifugation. The resuspended cells were disrupted by ultrasonication and inclusion bodies were collected by centrifugation and stirred overnight in 20 mM Tris pH 7.3, 200 mM NaCl, 8 M urea. The suspension was centrifuged at 18000 rpm for 1 h at 4 °C and the solubilized fraction loaded by gravity flow onto a nickel-bound chelating Sepharose resin (GE Healthcare) column. The column was washed with 20 mM Tris pH 7.3, 200 mM NaCl, 8 M urea and 20 mM imidazole, protein eluted with 8 M urea, 20 mM Tris, pH 7.3, 200 mM NaCl and 400 mM imidazole and refolded by dialysis with 20 mM Tris, pH 7.0, 200 mM NaCl, 0.5 M L-Arg, 0.4% protease inhibitor cocktail (Thermo Scientific, Pierce Protease and Phosphatase Inhibitor Mini Tablets, A32959) and 5 mM EDTA for 2 days. Gel filtration size exclusion chromatography was performed on Superdex 75 (GE Healthcare) in 20 mM bis-tris pH 6.6, 150 mM NaCl, 1 mM TCEP, 0.4% protease inhibitor cocktail and 1 mM EDTA.

#### 2.7.2 Nanobody-161 VHH production

The full-length Nanobody-161 gene sequence in the form of IgG1 was subcloned into vector, GSV0, which was modified to ensure secretion by ExpiCHO system cells. The nanobody was purified via Protein A resin and Superdex 200 running in buffer (20 mM Tris-HCl, pH 8.0, 150 mM NaCl). The nanobody was enzymatically digested with papain to remove the Fc region to allow complex formation (**Supplementary Table S1**).

#### 2.7.3 Antigen-antibody complex production

Purified TNFR2 and Nanobody-161 VHH were mixed in 1:2 molar ratio and the complex purified by gel filtration in 20 mM bis-Tris pH 6.6 containing 150 mM NaCl, 1 mM TCEP, 0.4% cocktail and 1 mM EDTA.

### 2.8 Bio-layer interferometry (BLI) analysis

BLI analyses were performed at 25 °C using Gator biosensor system (Probe Life) with ProA Biosensors (LN 2207023T28). Briefly, 30 nM Nanobody-161 IgG1 and 9 mutants with single amino acid mutations, R29A, F30G, R53A, S101A, Q102A, L103G, Y105G, F107G and R108A, in Q Buffer (PBS containing 0.02% Tween 20 and 0.2% BSA) were immobilized on Protein A biosensors for 120 s and 4800 nM-300 nM TNFR2-His protein in Q buffer added. Association was performed over 120 s and dissociation over 180 s. All binding sensorgrams were globally fit to a 1:1 Langmuir binding model. Data were further analyzed using GraphPad Prism 8.0 software and curves fitted with R^2^ > 0.95.

### 2.9 Crystallization and data collection

The nanobody-161 VHH/TNFR2 complex was concentrated to 12.3 mg/mL and crystallization screens carried out at 16° using sitting-drop vapor diffusion and commercially available buffer sets. Diffraction-quality crystals were obtained from 0.1 M sodium citrate, pH 5.5, containing 12.5% (w/v) PEG 6000 and the crystal optimized by adjusting concentrations of salt, precipitant and buffer pH. Crystals were flash-cooled in liquid nitrogen with 25% glycerol for cryoprotection. Data were collected on the BL02U beamline at the Shanghai Synchrotron Research Facility. The intensities measured were weak and diffraction was extended to 3.0 Å resolution. Three wedges of consecutive datasets were collected from the same crystal, each covering 60°, and merged. Crystals showed diffraction consistent with the space group, P212121 (a = 95.72 Å, b = 197.42 Å, c = 148.06 Å), and data set was integrated and scaled with HKL2000 (38).

### 2.10 Structure determination and refinement

Molecular replacement was performed by maximum likelihood using PHASER in Phenix suite to allow prediction of complex structure. Manual building and structure adjustments were performed in COOT and structures refined by PHENIX (39, 40).

### 2.11 Viability assays

Cultured huTNFR2-Jurkat cells were collected, centrifuged at 300 g, resuspended in complete culture medium, counted, 50 μL per well of a 2 × 10^5^ cells/mL suspension plated into 96-well round-bottom plates (Corning, 7007) and 25 μL serially diluted antibodies added per well with incubation at 37 °C for 2 h. 25 μL/well TNFα-Fc fusion protein dimer was added, plates incubated at 37 °C for 24 h and 50 μL/well Cell-Titer Glo (Promega, G7572) added with incubation for 10 min before luminescence detection by microplate reader (Molecular Devices, SpectraMax i3x). CellTiter 96^®^ AQueous One Solution Cell Proliferation Assay (MTS) (Promega, G3581) was also used for assessment of cell viability for Jeko-1 and huTNFR2-Jurkat cells.

### 2.12 Assays of Treg proliferation

Peripheral blood mononuclear cells (PBMCs) were isolated from whole blood by density gradient centrifugation and CD4+ T cells isolated using a kit (Miltenyi Biotec, 130-096-533), according to the manufacturer’s instructions. CD4+ T cells were centrifuged at 300 g, resuspended in complete medium, counted, 100 μL per well of a 2 × 10^6^ cells/mL cell suspension plated into 96-well plates (Corning, 3599) and 100 μL 400 U/mL IL2 (Novoprotein, CP09), 40 ng/mL TNFα (ACROBiosystems, TNA-H5228), Nanobody-161 and control antibody, OPI-SBT002e, added with incubation at 37 °C for 72 h. Cells were washed three times with FACS buffer and stained with PE-labeled anti-human CD4 flow cytometry antibody (1:200, BioLegend, 357404) and FITC-labeled anti-human CD25 flow cytometry antibody (1:200, BioLegend, 356106) at 4 °C for 30 min. Plates were washed three times with FACS buffer, fixed in 4% paraformaldehyde at room temperature for 30 min, washed three times with 1 x Perm solution and Alexa Fluor^®^ 647-labeled anti-human Foxp3 flow cytometry antibody (1:200, BioLegend, 320114) added with incubation at room temperature for 1 h. Plates were washed three times with FACS buffer, cells resuspended and labeled antibodies detected by flow cytometry (Beckman Coulter, CytoFLEX AOO-1-1102).

### 2.13 Assays of CD8+ T cell activation

96-well plates (Corning, 3370) were coated with 1 μg/mL anti-CD3 antibody and serially diluted Nanobody-161 and OPI-SBT002e added with incubation overnight. PBMCs were isolated from whole blood, as above, and CD8+ T cells isolated using a kit (Miltenyi Biotec, 130-096-495). 96-well plates were washed three times with PBS, 150 μL 10% FBS added and plates incubated for 2 h at 37 °C before washing 3x with PBS. CD8+ T cells were centrifuged at 300 g, resuspended in complete medium, counted and 100 μL per well of a 1 × 10^6^ cells/mL suspension added to the pre-blocked 96-well plates for incubation at 37 °C for 72 h. IFN-γ was measured in the conditioned medium with ELISA kit (BD, 555142), reading OD450 by microplate reader (SpectraMax 190).

### 2.14 Assays of ADCC

50 μL/well aliquots of 2 × 10^5^ cells/mL suspensions of huTNFR2-HEK293 or huJurkat-HEK293 cells were plated into 96-well round bottom plates (Corning, 7007) and 50 μL per well serially diluted Nanobody-161 or control antibodies, SIM-0235–001 and BI-1808, added for incubation at 37 °C for 20 min. For ADCC, PBMCs (Allcells, NF0074) were chosen as the effector cells instead of NK cells to mimic the physiological conditions using an E:T ratio of 2.5:1. Then, PBMCs were rested overnight, 50 μL per well of a 5 × 10^5^ cells/mL suspension added to the plates for incubation at 37 °C for 4 h. 60 μL per well LDH detection reagent (Beyond, C0017) was added and plates incubated at room temperature for 1 h before OD490 was read by microplate reader (SpectraMax 190) and cell death rate calculated.

### 2.15 TNFR2 humanized mouse tumor model

Humanized TNFR2 female C57BL/6 mice (8 weeks old) were purchased from Shanghai Southern Model Biotechnology Co., Ltd. 1.5 × 10^6^ cells of the mouse colon cancer cell line, MC38 (Institute of Basic Medical Sciences, Chinese Academy of Medical Sciences), were subcutaneously injected in the hind flank on Day 0. Mice were intraperitoneally injected with Nanobody-161, OPI-SBT002e or PBS on day 7 and twice a week for a total of 6 times. Antibody dosages were calculated for the 78 kDa Nanobody-161 at 7.50 mg per kg body weight (mpk) compared with the 150 kDa OPI-SBT002e at 14.42 mpk (2.50 mpk Nanobody-161 is equivalent to 4.81 mpk OPI-SBT002e and 0.50 mpk Nanobody-161 is equivalent to 0.96 mpk OPI-SBT002e). Body weight and tumor size were recorded weekly until tumors reached 1500 mm^3^ in the PBS control group. Tumor size was measured using digital calipers and volume calculated by the formula: L × W^2^/2, where L is the longest and W the shortest tumor diameter (mm). Serum was collected after the 4th and 5th antibody/PBS dose and ALT (njjcbio, C009-2-1) and AST (njjcbio, C010-2-1) measured using kits.

### 2.16 Statistical analysis

All experiments were repeated three times. Data are presented as mean ± standard deviation (SD) or standard error of the mean (SEM), as indicated. Statistical analyses were performed using GraphPad Prism 7.05 software (GraphPad Software, Inc.). Kruskal-Wallis non-parametric test followed by Dunn’s *post-hoc* test was used for multiple comparisons with a value of *P* < 0.05 considered to indicate statistical significance.

## 3 Results

### 3.1 Characterization of Nanobody-161

**Figure 1:**
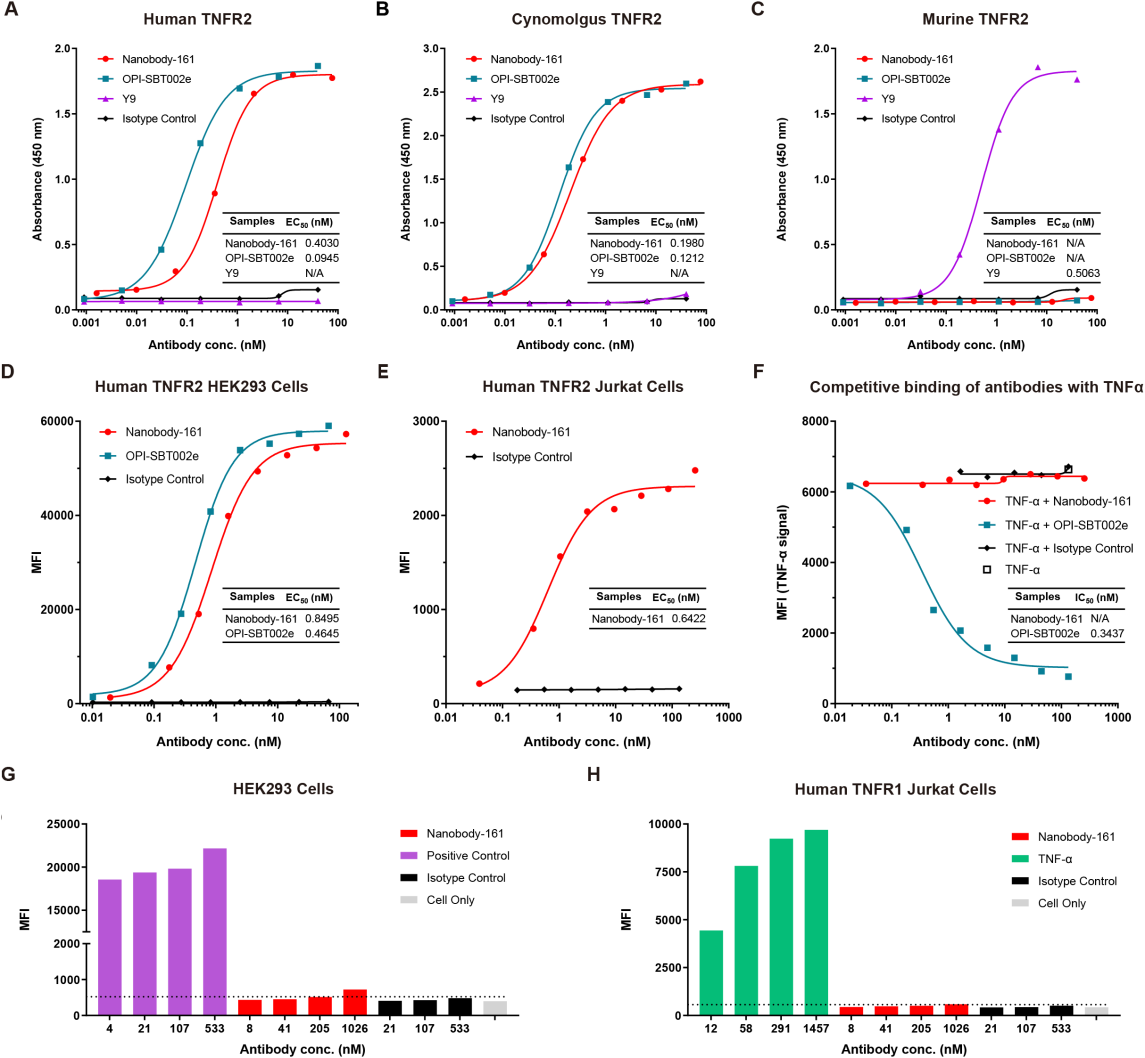
Characterization of Nanobody-161 by binding assays. **(A)** Binding of Nanobody-161 to human TNFR2 determined by ELISA (EC50 = 0.403 nM). Positive control antibody, OPI-SBT002e, and negative control, Y9, are shown. **(B)** Binding of Nanobody-161 to cynomolgus TNFR2 by ELISA (EC50 = 0.198 nM). Positive control antibody, OPI-SBT002e, and negative control, Y9, are shown. **(C)** Binding of Nanobody-161 to murine TNFR2 by ELISA. Positive control antibody, Y9, and negative control, OPI-SBT002e, are shown. **(D)** Binding of Nanobody-161 to human TNFR2-overexpressing HEK293 cells (huTNFR2-HEK293) by FACS (EC50 = 0.850 nM). Isotype control refers to native untransfected HEK293 cells. **(E)** Binding of Nanobody-161 to human TNFR2 overexpressing Jurkat cells (huTNFR2-Jurkat) by FACS (EC50 = 0.642 nM). Isotype control refers to native untransfected Jurkat cells. **(F)** Competitive inhibition assay with both Nanobody-161 and TNFα to show binding to huTNFR2-HEK293 cells. **(G)** Binding of Nanobody-161 to huTNFR2-HEK293 cells by FACS. Positive control: IgG1 which was known to bind HEK293 cells; isotype control: IgG1. **(H)** Binding of Nanobody-161 to Jurkat cells overexpressing human TNFR1 by FACS. Positive control: Anti-TNFR1 antibody; isotype control: IgG1. All experiments were repeated three times. Data are presented as mean ± SD or SEM. TNFR2, Tumor necrosis factor receptor 2; ELISA, Enzyme linked immunosorbent assay; MFI, mean fluorescence intensity; FACS, Fluorescence-activated cell sorting; SD, standard deviation; SEM, standard error of the mean.

A novel humanized nanobody, Nanobody-161, that binds TNFR2 was identified using alpaca immunization and phage display. Binding to human, cynomolgus and murine recombinant TNFR2 was tested by ELISA (**Figures 1A-C**) relative to the humanized anti-human TNFR2 antibody, OPI-SBT002e, and anti-mouse TNFR2 antibody, Y9 (20, 41). Nanobody-161 bound human and cynomolgus TNFR2 but was not observed to bind murine TNFR2, consistent with the high level of sequence homology (97%) between the human and cynomolgus TNFR2 extracellular domain (ECD) and the low level (56%) between human and murine TNFR2-ECD from Uniprot database. Nanobody-161 also bound strongly to HEK293 and Jurkat cells overexpressing human TNFR2 (huTNFR2-HEK293 and huTNFR2-Jurkat) but not to the native untransfected cells (**Figures 1D, E**) and it also was not observed to bind Jurkat cells overexpressing the alternative TNFα-binding receptor, TNFR1 (**Figure 1H**). Nanobody-161 was not observed to bind other TNFR superfamily members, CD27, GITR, 4-1BB, OX40, CD40, BCMA, RANK and DR3 (**Supplementary Figure S1**). Experiments to evaluate the competition between Nanobody-161 and TNFα for TNFR2 binding showed that Nanobody-161 was not observed to block TNFα binding in huTNFR2-HEK293, unlike the conventional antagonist, OPI-SBT002e (**Figures 1F, G**). BI-1808, SIM-0235–001 and LBL-019 were all shown to be antibodies that blocked TNFα binding and HFB200301 was not observed to, consistent with previous reports (28, 29, 42, 43) (**Supplementary Figure S2**). These results indicate that Nanobody-161 is specific for TNFR2.

### 3.2 Structural Analysis of the Nanobody-161 VHH/TNFR2 complex

**Figure 2:**
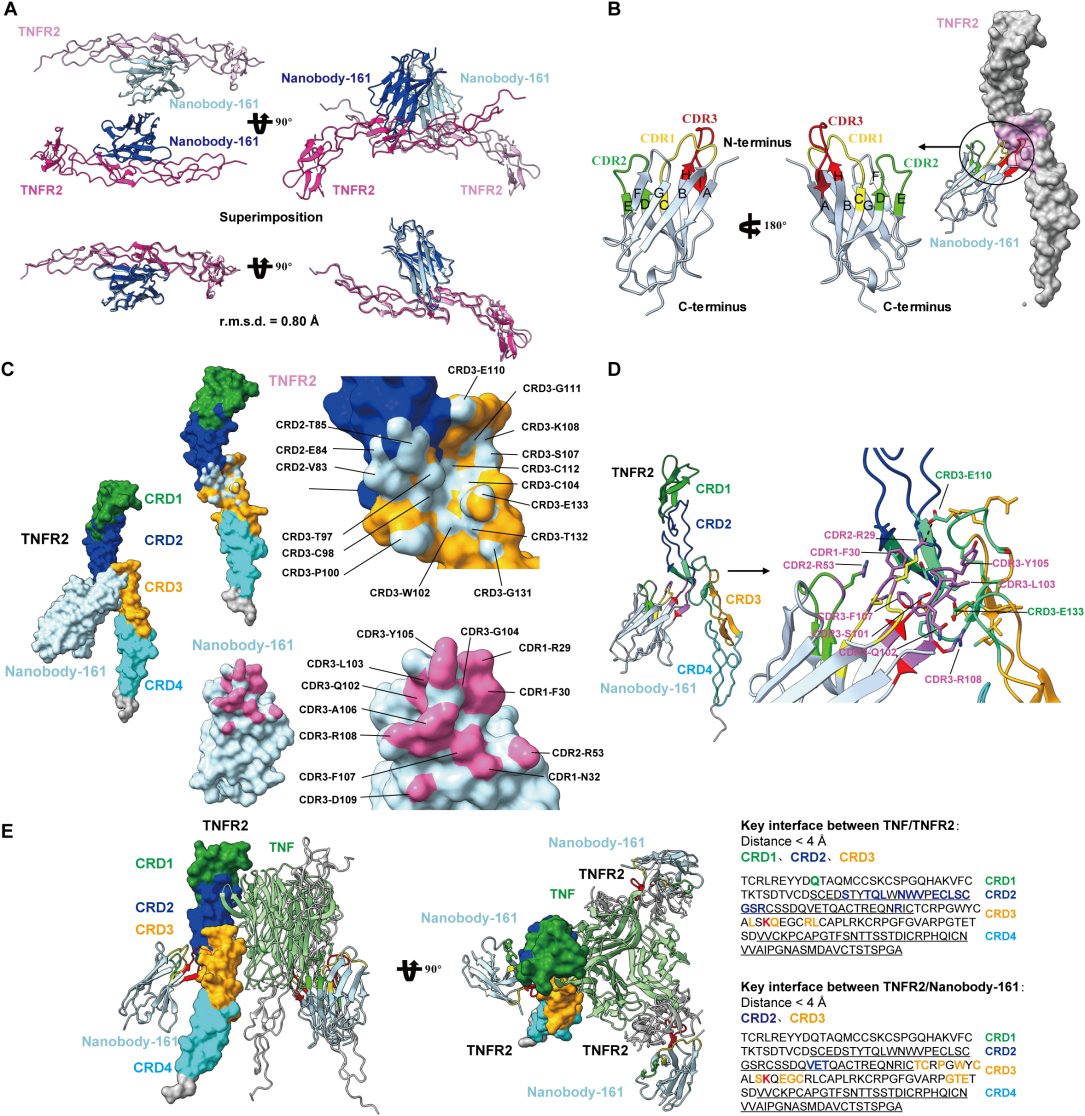
Crystal structures of Nanobody-161 in complex with TNFR2. **(A)** Ribbon diagrams of the asymmetric unit of TNFR2 and Nanobody-161 complex in space group P212121. **(B)** Ribbon representation of Nanobody-161 showing the composition of 9 β-sheets from A to l CRD1: yellow; CRD2: green; CRD3: red. **(C)** Surface analysis of key residues involved in TNFR2 and Nanobody-161 interaction. TNFR2 epitope shown in light blue; Nanobody-161 paratope shown in pink. **(D)** Interface analysis showing TNFR2/Nanobody-161 interactions, including salt bridge, hydrogen bonds and hydrophobic interactions. TNFR2 CRD1: green; CRD2: blue; CRD3: orange; CRD4: cyan. Nanobody-161 CDR1: yellow; CDR2: green; CDR3: red. **(E)** Superimposition of Nanobody-161/TNFR2 and TNFα/TNFR2 complexes. Structure of the TNFα/TNFR2 complex was derived from the (PDB ID: 3ALQ). Left panel: TNFα: green; TNFR2: cyan; Nanobody-161: red. Overlapping regions are shown in yellow. Right panel: key interface residues in TNFR2. Colored residues participate in TNFα or Nanobody-161 binding. The residue shown in red participated in both interactions. TNFR2, Tumor necrosis factor receptor 2; PDB, protein data bank.

**Figure 3:**
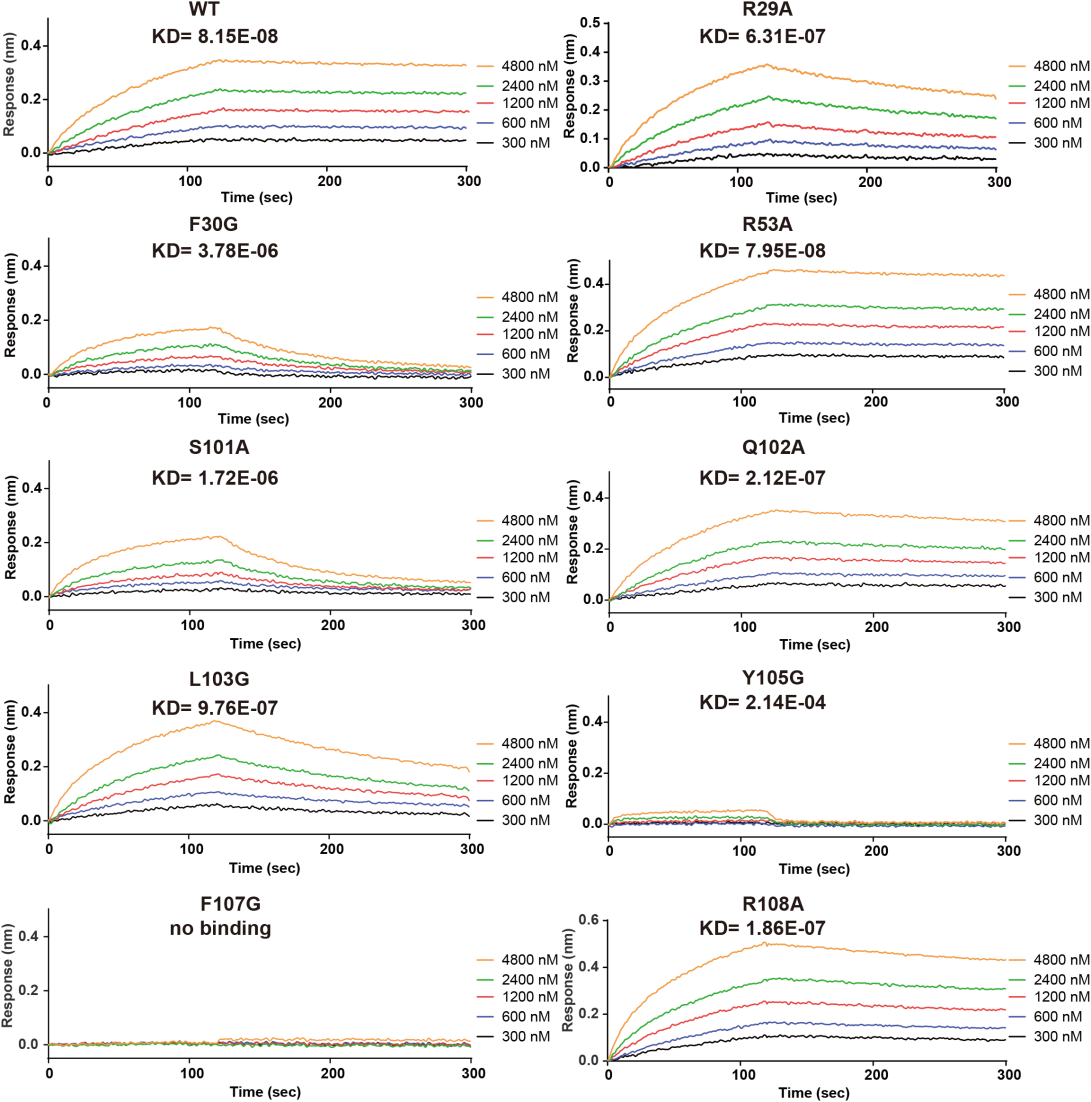
Site-directed mutagenesis of key Nanobody-161 residues interacting with TNFR2. Binding and dissociation curves for Nanobody-161 WT and mutants R29A, F30G, R53A, S101A, Q102A, L103G, Y105G, F107G and R108A binding to histidine-tagged TNFR2-.TNFR2, Tumor necrosis factor receptor 2; WT, wild type.

**Figure 4:**
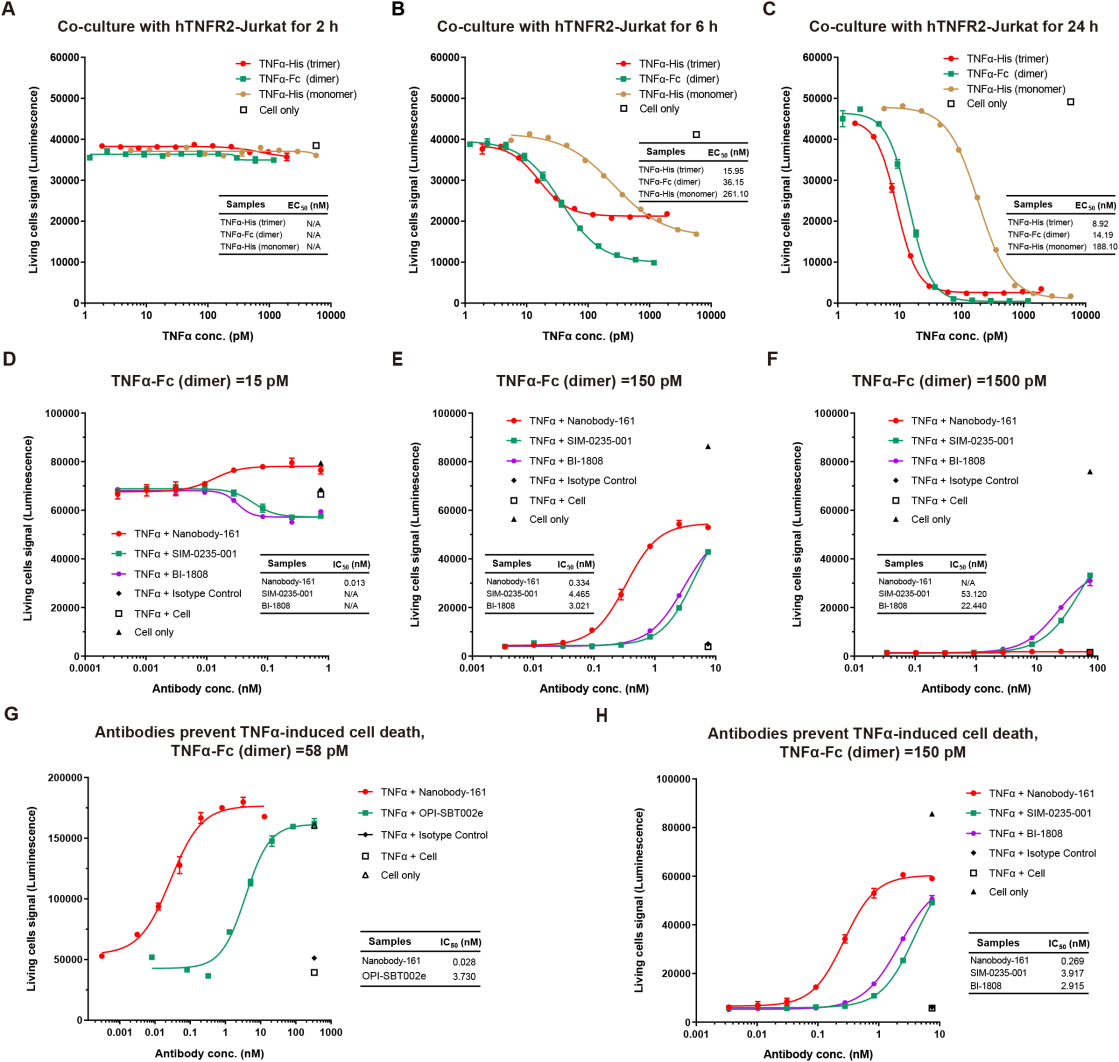
Effects of Nanobody-161 on apoptosis in huTNFR2-Jurkat cells treated with TNFα. **(A–C)** Dose-response curves for TNFα-mediated cell death of hTNFR2-Jurkat cells at different time points. **(D–F)** Dose-response curves for Nanobody-161 and cell death in huTNFR2-Jurkat cells pre-incubated with 15pM, 150pM and 1500pM TNFα for 5 min. **(G)** Dose-response curve for Nanobody-161 and cell death in huTNFR2-Jurkat cells pre-incubated with 58 pM TNFα for 30 min. **(H)** Dose-response curve for Nanobody-161 and cell death in huTNFR2-Jurkat cells pre-incubated with 150 pM TNFα for 30 min. TNFR2, Tumor necrosis factor receptor 2.

**Figure 5:**
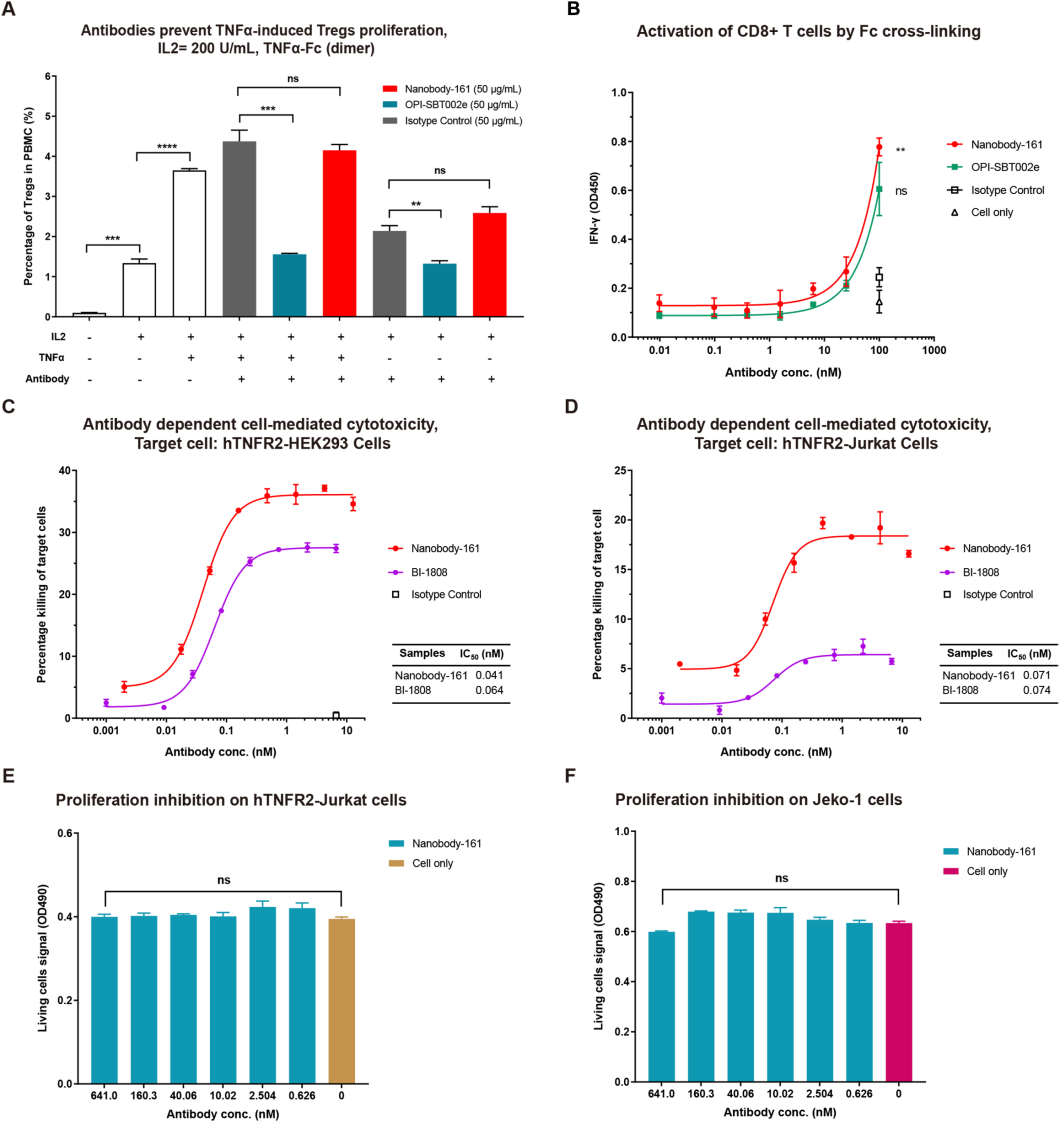
Nanobody-161 effects on Tregs, CD8+ T cells and huTNFR2-expressing cells. **(A)** Effects of Nanobody-161 on TNFα-induced proliferation of Tregs in PBMCs. Isotype control: IgG1 targeting an irrevalent target except TNFR2. **(B)** Effects of Nanobody-161 on CD8+ T cell activation and IFN-γ secretion *in vitro*. **(C, D)** Effects of Nanobody-161 on ADCC in huTNFR2-HEK293 and huTNFR2-Jurkat cells. **(E, F)** Effects of Nanobody-161 on proliferation of huTNFR2-Jurkat and human tumor Jeko-1 cells. TNFR2, Tumor necrosis factor receptor 2; PBMCs, peripheral blood mononuclear cells; ADCC, antibody-dependent cell-mediated cytotoxicity; Tregs, T cells. **P < 0.01, ***P < 0.001, ****P < 0.0001.

The crystal structure of the complex formed between residues 23–257 of the human TNFR2 and residues 1–121 of the VHH region of Nanobody-161 was determined by X-ray diffraction and refined to a 2.9 Å resolution with R-work of 20.3% and R-free of 27.0%. The crystal structure belongs to space group, P212121, with two independent copies of the complex in the asymmetric unit, “H”-shaped organization and superpose with a Cα-root-mean-square deviation (Cα-rmsd) of 0.80 Å (**Figure 2A**). The Nanobody-161 VHH fragment bound to the groove formed by TNFR2 CRD2 (residues 55-96) and CRD3 (residues 97-140). CRD1 (residues 17-54) and CRD4 (residues 141-178) are not involved in the interaction. The VHH fragment was seen to consist of nine antiparallel β-sheets with planes formed by A, B, F, G and C, D, E, H, I and the CDR3 loop inserts into the cavity formed by TNFR2 CRD2 and CRD3 (**Figure 2B**). Interface analysis revealed an area of 662.9 Å2 composed of almost all residues in Nanobody-161 CDR3 (residues 99-110), some residues from CDR2 (residues 50-59) and some from CDR1 (residues 26-35) (**Figures 2B**-**5D**).

The Nanobody-161 VHH-TNFR2 structure was superimposed on the TNFα-TNFR2 structure (PDB ID: 3ALQ) to highlight ligand receptor interaction characteristics (44). TNFα and Nanobody-161 were found to interact with different sides of the TNFR2 extracellular face (**Figure 2E**) which explains why Nanobody-161 was not observed to block TNFα binding. The trimeric form of TNFα binds to the CRD1, CRD2 and CRD3 regions of TNFR2 while Nanobody-161 binds to the CRD2 and CRD3 regions. CRD3 residue, Lys108, was involved in both interactions and may be crucial for downstream signal transduction.

### 3.3 Interaction of Nanobody-161 VHH with TNFR2

Site-directed mutagenesis of Nanobody-161 IgG1 was performed and binding affinity constants (KD) measured for each mutant by BLI assays (**Figure 3**). Interface analysis showed salt bridges, hydrogen bonds and hydrophobic interactions to be involved in the Nanobody-161/TNFR2 interaction (**Supplementary Table S2**). Salt bridges were formed between residue Glu110 in the TNFR2 CRD3 region and residue Arg29 in the Nanobody-161 VHH CDR1 region. Nanobody-161 VHH CDR1 residue Arg53 and CDR3 residues Ser101, Gln102, Leu103, Gly104, Tyr105, Ala106, Phe107 and Arg108 formed hydrogen bonds with TNFR2 CRD3 residues Thr97, Cys98, Lys108, Gly131, Thr132 and Glu133 and CRD2 residue Glu84. Hydrophobic interactions involved Nanobody-161 VHH CDR3 residues Phe30, Ala106 and Phe107 and TNFR2 CRD3 residues Val83, Ile95 and Pro100. F30G, S101A and Y103G had the greatest impact on Kd values, indicating the significance of hydrogen bonding and hydrophobic interactions in mediating binding (**Supplementary Table S3**).

### 3.4 Inhibition of TNFα-induced apoptosis by Nanobody-161

A monomeric form of TNFα with His tag, dimeric form with Fc tag and trimeric form with His tag were tested for binding to huTNFR2-Jurkat and apoptosis occurred within 6–24 h (**Figures 4A-C**). No cell death was observed within the first 2h. Nanobody-161 inhibited TNFα-TNFR2 signaling in huTNFR2-Jurkat cells pre-incubated with antibodies for 5 minutes before 15 pM to 1500 pM TNFα was added. 84% hTNFR2-Jurkat cells survived at 15 pM TNFα in the absence of Nanobody-161 and 100% survived with 0.1–1 nM Nanobody-161 (**Figure 4D**). Indeed, Nanobody-161 showed approximately ten-fold increased potency over conventional antagonists at 150 pM TNFα (**Figure 4E**). However, Nanobody-161 showed little inhibitory activity at a 1500 pM TNFα, 10–100 times higher than normal physiological levels, although conventional antagonists did retain some capacity to reduce apoptosis (**Figure 4F**). HuTNFR2-Jurkat cells were pre-incubated with antibodies for 30 minutes before physiological concentrations of 58 pM and 150 pM TNFα were added (**Figures 4G, H**). All antibodies, including the non-blocking Nanobody-161 and blocking OPI-SBT002e, SIM-0235-001 (45) and BI-1808 (46) antagonists, prevented apoptosis with Nanobody-161 showing the most potent effects.

### 3.5 Nanobody-161 promotes Treg proliferation and CD8+ T cell activation, stimulates ADCC

CD4+ T cells were purified from PBMCs, co-cultured with IL2, TNFα and antibodies and the proportion of Tregs measured after 72 h. TNFα stimulated Treg proliferation by 3.65% compared with 1.33% for IL2 alone, consistent with the activation of nuclear factor κB in these cells (15). OPI-SBT002e suppressed Treg proliferation in the presence of TNFα, producing a rate of 1.56% (**Figure 5A**). Nanobody-161 was not observed to affect Treg proliferation, yielding a rate of 4.15% in the presence of TNFα (**Figure 5A**). Human CD8+ T cells were stimulated with 1 μg/mL anti-CD3 antibody and 0.5 μg/mL anti-CD28 antibody in the presence of increasing concentrations of Nanobody-161. Nanobody-161 enhanced the activation of CD8+ T cells and increased IFN-γ secretion (**Figure 5B**). NK cell-mediated cytotoxicity against huTNFR2-HEK293 and huTNFR2-Jurkat cells was assessed by LDH release into the medium. Nanobody-161 showed a maximum ADCC effect of 37.1% for huTNFR2-HEK293 and 19.2% for huTNFR2-Jurkat cells and was more potent than the control BI 1808 antibody. The lower ADCC activity for huTNFR2-Jurkat cells may be explained by the lower TNFR2 expression by this cell-type (**Figures 3D**–**5C**). HuTNFR2-Jurkat and Jeko-1 cells were cultured with varying concentrations of Nanobody-161 and cell survival assessed by MTS assays after 72 h. Nanobody-161 alone was not observed to induce apoptosis (**Figures 3F**-**5F**). Thus, Nanobody-161 may stimulate the anti-tumor activity of immune cells in the TME, enhance the ADCC effect in tumors, and was not observed to directly induce tumor cell death which make it a promising candidate for cancer therapy.

### 3.6 Nanobody-161 had anti-tumor effects in a transgenic mouse model

**Figure 6:**
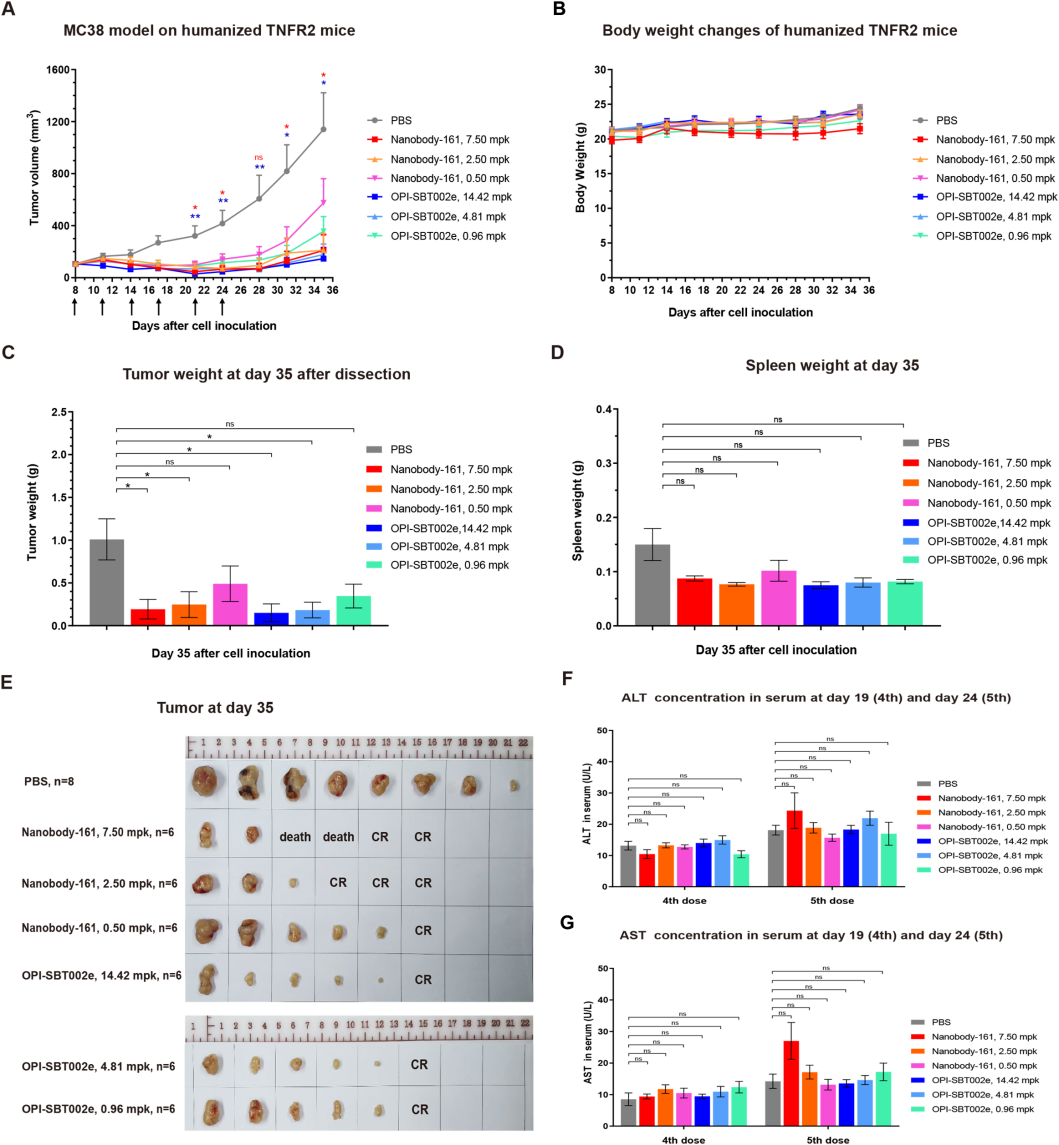
The efficacy of Nanobody-161 in transgenic mouse model. **(A)** Effects of 0.5, 2.5 and 7.5 mpk Nanobody-161 on tumor volume over time in a mouse human TNFR2-expressing model of colon adenocarcinoma generated with MC38 cells. **(B)** Effects of 0.5, 2.5 and 7.5 mpk Nanobody-161 on body weight over time in a mouse model of colon adenocarcinoma generated with MC38 cells. **(C)** Effects of 0.5, 2.5 and 7.5 mpk Nanobody-161 on tumor weight on Day 35 (tumor weight was plotted as 0.01 g). **(D)** Effects of 0.5, 2.5 and 7.5 mpk Nanobody-161 on spleen weight on Day 35. No detectable spleen enlargement was found. **(E)** Photos of tumors dissected on Day 35. **(F, G)** Levels of serum ALT and AST on Day 19 (4th dose) and Day 24 (5th dose). TNFR2, Tumor necrosis factor receptor 2; CR, complete regression; ALT, alanine aminotransferase; AST, aspartate aminotransferase. *P < 0.05, **P < 0.01.

TNFR2-humanized mice were subcutaneously inoculated with MC38 tumor cells and treated with antibodies for 35 days when tumors reached 80 mm^3^ to allow comparison of the effects of Nanobody-161 and OPI-SBT002e. Both OPI-SBT002e and Nanobody-161 inhibited tumor growth without producing adverse side effects (**Figures 4B**-**6A**). 7.50 mpk Nanobody-161 had similar tumor inhibitory activity to 14.42 mpk OPI-SBT002e, as did 2.50 mpk Nanobody-161 to 4.81 mpk OPI-SBT002e. Weaker effects were seen by 0.50 mpk Nanobody-161 and 0.96 mpk OPI-SBT002e. Two mice in the 7.50 mpk Nanobody-161 dosage group died on Days 24 and 28 following the 6th dose (**Supplementary Table S4**), perhaps due to excessive peripheral blood Treg depletion caused by ADCC. Medium dose 2.50 mpk Nanobody-161 showed a complete regression (CR) ratio of 50% (3/6) which was greater than that of the equivalent 4.81 mpk OPI-SBT002e dose. Assessment of tumor weight (**Figure 6C**), spleen weight (**Figure 6D**) and tumor morphology (**Figure 6E**) on day 35 showed no enlargement of the spleen as tumor growth was inhibited. Serum levels of ALT and AST measured after the 4th and 5th administrations showed no significant differences with Nanobody-161 treatment indicating no adverse impact on liver function (**Figures 4G**–**6F**).

## 4 Discussion

A novel nanobody, Nanobody-161, was identified and introduced as a potential candidate for cancer therapy in the current work. Nanobody-161 was found to enhance CD8+ T cell activation and induce IFN-γ secretion by Fc crosslinking and stimulated ADCC both *in vitro* and *in vivo*. Bispecific (bsAb) or trispecific antibody (tsAb) assembly is more accessible using Nanobody-161 than IgG. These properties contribute to making Nanobody-161 a promising candidate for anti-cancer therapy since it is expected to have an impact on tumor growth without causing immunosuppression.

The role of TNFR2 in mediating the functions of anti-tumor effector T cells and inhibitory Tregs makes it a suitable target for anti-cancer therapy (13, 14). Agonistic antibodies to boost effector T cell activity or antagonistic antibodies to inhibit Tregs have entered clinical trials. Five drugs composed from three agonistic antibodies, LBL-019 (Phase 1/2) (47), BI-1910 (Phase 1/2) (48) and HFB200301 (Phase 1) (29) and two antagonistic antibodies, BI-1808 (Phase 2) (42) and SIM-0235-001 (Phase 1) (43), have been the subject of preliminary clinical readouts. No dose-limiting toxicity was observed and the MTD (Maximum Tolerable Dose) was not reached, indicating good safety for targeting TNFR2. Efficacy data show partial response (PR) or stable disease (SD) as the best clinical response achieved by monotherapy but combination therapy with PD-1/L1 inhibitors is ongoing. Anti-TNFR2 agonistic antibodies have also been used to selectively kill autoreactive T cells and treat autoimmune diseases, such as type 1 diabetes, in preclinical studies (27). Besides, AN3025, a partially blocking antagonistic rabbit antibody showed a decreased Tregs numbers and increased IFN-gamma and Granzyme expression via RNA-seq of mice tumors (49). The paratope in agnostic and antagonistic antibody could make a difference on the effects towards Tregs and effector T cells.

Recently, BioInvent has paused agonist BI-1910 development to focus resources on its more advanced antagonist BI-1808 program following a strategic review despite positive safety results from BI-1910. For BI-1808, it blocks TNFα binding and exerts FcγR-dependent depletion of Tregs. Phase 2 results from 9 cutaneous T cell lymphoma (CTCL) patients showed an ORR (Objective response rate) of 45% and a DCR (Disease control rate) of 100%. Especially, there was 1 CR (Complete response), 3 PR (Partial response), and 5 SD (Stable disease). All treatment related adverse events were classified as mild or moderate with no potentially related Grade 3+ AE reported (50). As for BI-1910, it didn’t block TNFα binding but could promote the activation and proliferation of TNFR2 expressed NK and effector T cells. Preliminary Phase 1 results showed that Part A dose escalation has been completed and reached a biologically active dose level. Data show 50% stable disease (SD) as best clinical response with no notable adverse events even at the highest doses tested (51). Both drugs showed good safety in clinical trials, indicating the feasibility of targeting TNFR2. Despite different mechanism, the good efficacy of BI-1808 in CTCL provide a promising direction for treatment with such cold tumors by promoting CD8 infiltration and granzyme B production in patients. More clinical results from TNFR2 antibody will tell the priority from antagonist and agonist.

TNFR2 membrane receptors can be converted into soluble forms (sTNFR2) through the activity of TACE enzymes. sTNFR2 has been as a biomarker of kidney tissue damage and long-term renal outcome in lupus nephritis (52). The latest preclinical data on pancreatic ductal adenocarcinoma indicate that the combination of anti-TNFR2 and CD40 agonists demonstrates superior efficacy compared to monotherapy by eliminating activated Treg cells and alleviating CD8 depletion (53). In non-small cell lung cancer, combination therapy with PD-1 inhibitors, PTP1B inhibitors, and TNFR2 antibodies increased the proportion of CD8 T cells and reduced the proportion of Treg cells in the spleen, lymph nodes, and tumors of NSCLC models compared to monotherapy, effectively inhibiting tumor growth (54). A 2024 study indicates that TNFR2^+^ endothelial cells can modulate CD8 cell exhaustion and chemotherapy resistance (55). A 2025 study on hepatocellular carcinoma highlights the role of serum TNFR2 in risk stratification, suggesting that sTNFR2 may serve as a biomarker (56). Whether sTNFR2 could act as a sink effect need more preclinical study.

TNFα has been demonstrated to activate TNFR2 signaling by binding to the receptor’s CRD1 and CRD2 domains (4). The first reported Nanobody-161/TNFR2 crystal structure indicated that the CRD2 and CRD3 TNFR2 regions were involved in binding and these are different from the CRD1, CRD2 and CRD3 regions involved in TNFα/TNFR2 binding which explains why the two ligands form independent associations with the receptor. The involvement of CRD3 Lys108 in both interactions may explain the effect of Nanobody-161 in inhibiting TNFα-TNFR2 signaling. Antagonistic antibodies have previously been reported to lock the receptor in its conformationally inactive state (57). Two models of TNFα-TNFR2 signaling amplification have been presented incorporating either two or three TNFα trimers binding dimeric or trimeric TNFR2 molecules and generating an aggregate of TNFα-TNFR2 complexes on the cell surface (58). Traditional TNFα-TNFR2 signal pathway requires the oligomerization of TNFR2 to activate the downstream signal. However, a non-blocking Nanobody-161 might disrupt the oligomerization via direct binding without competition with ligand TNFα or have an influence on the conformational changes of TNFR2 ectodomian. We believe that the binding of the bivalent Nanobody-161 may interfere with the physiological assembly of the trimeric TNFα-TNFR2 complex and activation of intracellular signaling.

Human TNFα is thought to predominantly exist as a trimer (59). All forms of TNFα stimulated apoptosis in huTNFR2-Jurkat cells with the monomeric being less potent than the dimeric and trimeric forms. TNFα is considered to bind TNFR2 and initiate apoptosis with no discernible effect on cells lacking TNFR2 (41). The capacity of Nanobody-161 to inhibit TNFR2 signaling without interfering with TNFα binding may account for its superior performance. Non-blocking Nanobody-161 might lock the specific conformations or disrupt the oligomerization of TNFR2, resulting in blockade of downstream signal pathways involved in apoptosis relevant to TNFα binding. TNFR2 is predominantly expressed by Tregs (60) and expression increases with tumor progression (61), resulting in immunosuppression in the TME (62, 63). Antibody-mediated trimerization and activation of TNFR2 is thought to stimulate conventional T cells (64, 65). Dominant TNFR2 antagonists have been described which block the binding of TNFα, suppressing proliferation of Tregs, as have non-blocking antagonists which have a weaker effect and are outcompeted by high TNFα concentrations in binding assays (15). Nanobody-161 would fall into the category of a non-blocking antagonistic antibody using these criteria but nonetheless has potent antitumor activity.

The superior anti-tumor effects in a transgenic mouse model proved Nanobody-161 a good candidate for clinical study. Despite two mouse deaths in the high dose group of 7.50 mpk, no discernible toxicity was observed in other mice. Individual differences among mice may be the cause of death and excessive peripheral blood Treg depletion or potential off-target effects are also possible explanations. Previous animal studies analyzed the number and proportion of immune cells in tumors and spleens, such as CD3+ T cells, CD4+ T cells, CD8+ T cells and Tregs. However, due to potential early sampling time issues, no significant differences were detected (Data not shown). Therefore, more preclinical experiments in mouse models should be conducted for a better understanding of safety and MTD.

Immune checkpoint inhibitors (ICIs), including anti-PD-1, -PD-L1 and -CTLA-4 antibodies, have revolutionized cancer therapy (7). TNFR2 may be considered an immune checkpoint protein due to its high and exclusive expression on inhibitory Tregs and MDSCs (19). Combination cancer immunotherapy targeting TNFR2 and PD-1/PD-L1 has shown promising preclinical results in pancreatic ductal adenocarcinoma (PDAC) mouse models (66). Nanobody-161 inhibited tumor growth and enhanced ADCC effects without adverse side effects in a transgenic mouse model. We consider that Nanobody-161 may represent an addition to the canon of ICIs. The combination therapy has synergistic effects on release of Teff from intrinsic immune checkpoint brakes and clearing extrinsic obstacles from Tregs or MDSCs. Nanobody-161 in combination with ICIs could remodel the TME from “immunosuppressive desert” to “immune-permissive niche” and overcome potential ICI Resistance. ISM5939, an oral ENPP1 inhibitor, enhanced STING signaling and synergizes with PD-1, suggesting a rational triplet path (Nb-161 + PD-1 + ENPP1i) where TNFR2^+^ Tregs and myeloid suppression co-exist (67). Besides, preclinical study showed that combination of anti-TNFR2 mAb with agonistic anti-CD40 mAb promoted stronger T cell activation in PDAC mice models (53). This study open a new combination therapy of CD40/TNFR2 bispecific antibody, which could both boost antigen presentation in APCs and decrease the immune inhibition by Tregs. Combination of Nb-161 and CD40 might refuel the immune system and enhance T cell infiltration and killing effects.

Nanobody-161 showed good antitumor activity but its *in vivo* efficacy could be improved by structure-based affinity maturation. Future studies are planned to provide experimental evidence for the conformational inhibition mechanism. Further experiments involving CD40/TNFR2 bsAb, PD-1 (PD-L1)/TNFR2 bsAb or TAA (TSA)/PD-1 (PD-L1)/TNFR2 tsAb in combination with Nanobody-161 are also in prospect to optimize clinical results.

## 5 Conclusion

In conclusion, the present study introduces a novel nanobody, Nanobody-161, which binds to TNFR2 without impeding TNFα binding. The crystal structure of the Nanobody-161/TNFR2 complex has been solved and indicates that Nanobody-161 binds to a different site on TNFR2 from that bound by TNFα. Nanbody-161 inhibited TNFα signaling and reduced TNFα-stimulated apoptosis in HEK293 and Jurkat cells overexpressing TNFR2. Nanobody-161 inhibited tumor growth in a transgenic mouse model. We suggest that the non-blocking TNFR2-antogonist, Nanobody-161, is a suitable candidate for the development of an anti-cancer therapy. Future studies will be conducted on the combination of Nanobody-161 with other immunotherapies or traditional chemotherapy drugs to explore synergistic effects in treating different tumor types, offering cancer patients more effective treatment options. Furthermore, given Nanobody-161’s advantages in constructing bispecific or trispecific antibodies, future efforts may design more complex antibodies targeting multiple tumor-associated antigens to enhance treatment specificity and efficacy.

## Data Availability

The datasets presented in this study can be found in online repositories. The names of the repository/repositories and accession number(s) can be found in the article/**Supplementary Material**.
