# Supplementary material for: Novel nanobody-161 binds tumor necrosis factor receptor 2 (TNFR2) to exert an anti-tumor effect but does not block TNFα-binding
Source: Front Immunol. 2025 Dec 8;16:1694313. doi: 10.3389/fimmu.2025.1694313 (PMC12719470; doi:10.3389/fimmu.2025.1694313)
Supplement: Supplementary file 1 [file Table1.docx]

**Table 1. Data collection and refinement statistics.**

| **Data collection** |  |
| --- | --- |
| Space Group | P2_1_2_1_2_1_ |
| Cell dimensions |  |
| a, b, c (Å) | 63.10 96.48 122.70 |
| α, β, γ, (°) | 90 104.413 90 |
| Resolution (Å) | 37.66 - 2.95 |
| R_merge_ (%) | 87.2 (79.9) |
| I / σI | 9.67 (2.65) |
| Completeness (%) | 89.03 (56.03) |
| Redundancy | 3.6 (3.5) |
| **Refinement** |  |
| Resolution (Å) | 37.66 - 2.95 |
| No. reflections | 14898 |
| R_work_ / R_free_ (%) | 26.4/ 29.5 |
| No. atoms |  |
| Protein | 4339 |
| Ligands | 0 |
| solvent | 0 |
| B-factors | 58.87 |
|  |  |
| R.m.s. deviations |  |
| Bond lengths (Å) | 0.007 |
| Bond angles (°) | 1.15 |
| Ramachandran plot statistics (%) |  |
| Most favoured | 95.25 |
| Additional allowed | 4.75 |
| Generously allowed | 0.0 |
| Disallowed | 0.0 |

One crystal was used for each structure.

Values in parentheses are for the highest resolution shell. *Rmerge*=ΣhΣi|*Ih,i*-*Ih*|/ΣhΣi*Ih,i*, where *Ih* is the mean intensity of the *i* observations of symmetry related reflections of *h*. *R*=Σ|*Fobs*-*Fcalc*|/Σ*Fobs*, where *Fcalc* is the calculated protein structure factor from the atomic model (Rfree was calculated with 5.26% of the reflections selected randomly).
