# Supplementary material for: Novel nanobody-161 binds tumor necrosis factor receptor 2 (TNFR2) to exert an anti-tumor effect but does not block TNFα-binding
Source: Front Immunol. 2025 Dec 8;16:1694313. doi: 10.3389/fimmu.2025.1694313 (PMC12719470; doi:10.3389/fimmu.2025.1694313)
Supplement: Supplementary file 2 [file Table2.docx]

Supplementary Material

# Supplementary Data

Fig. S1. Nanobody-161 did not bind to other TNFR superfamily members.

Fig. S2. Characterization of clinical antibodies targeting TNFR2 in binding and competitive binding assays.

Table S1. Drug developability of Nanobody-161 antibody.

Table S2. Survival rates in MC38 xenograft humanized TNFR2 mice model.

Table S3. Key interactions involved in TNFR2-Nanobody-161 complex.

Table S4. Summary of the binding affinity constant (KD) of Nanobody-161 WT and mutants interacting with TNFR2-His.

# Supplementary Figures and Tables

## Supplementary Figures


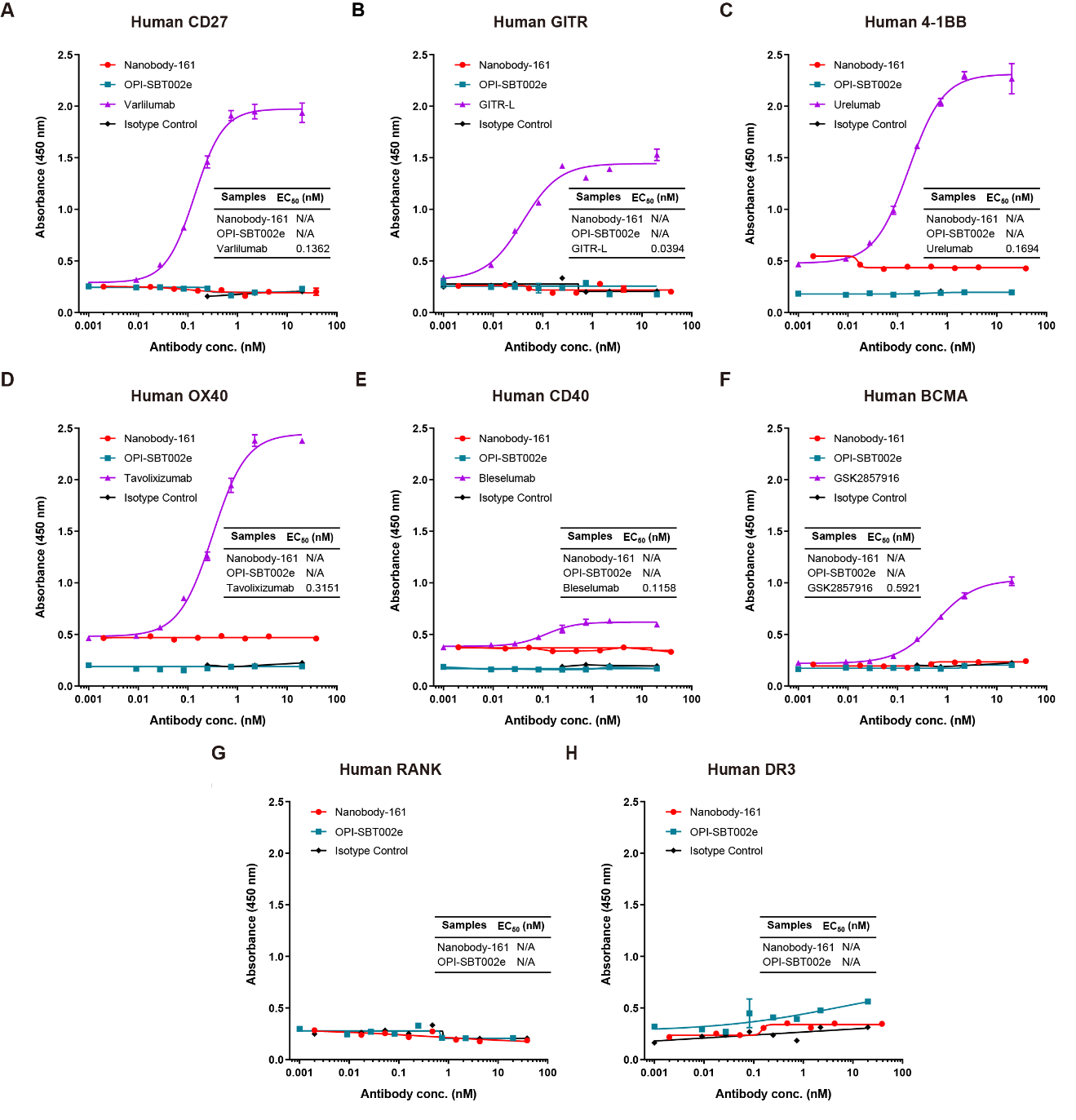


**Supplementary Figure 1.** ELISA assays of binding of Nanobody-161 to members of TNFR superfamily. (A) Human CD27. (B) Human GITR. (C) Human 4-1BB. (D) Human OX40. (E) Human CD40. (F) Human BCMA. (G) Human RANK. (H) Human DR3. Abbreviations: TNFR: Tumor necrosis factor receptor; ELISA: Enzyme linked immunosorbent assay.


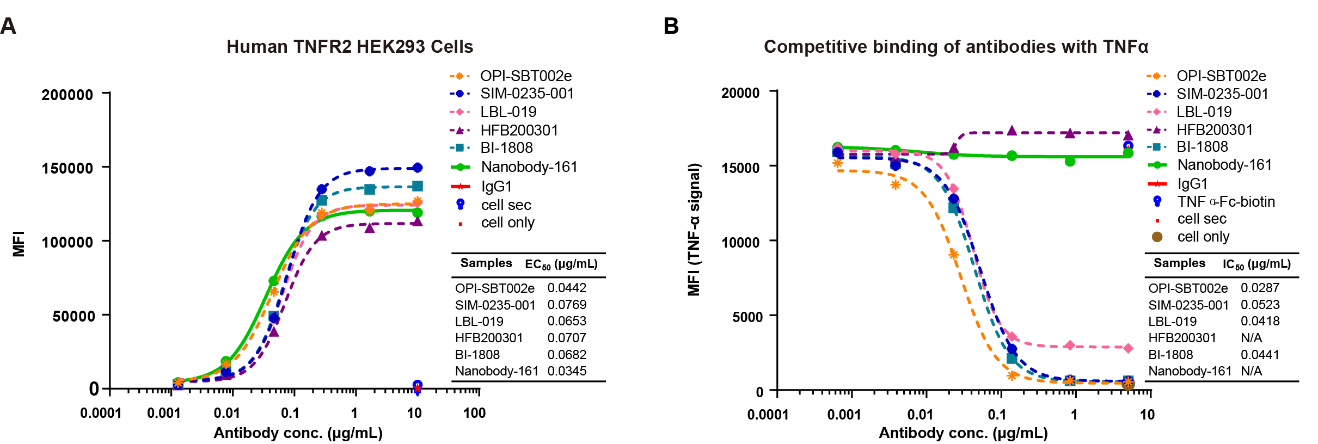


**Supplementary Figure 2.** Binding and blocking assays of clinical antibodies targeting TNFR2. (A) Binding of OPI-SBT002e, SIM-0235-001, LBL-019, HFB200301, BI-1808, Nanobody-161 and IG1 control to human TNFR2-overexpressing HEK293 cells by FACS. (B) Competitive assay to show inhibition of OPI-SBT002e, SIM-0235-001, LBL-019, HFB200301, BI-1808, Nanobody-161 and IgG1 control towards TNFα binding to human TNFR2-overexpressing HEK293 cells. Abbreviations: TNFR2: Tumor necrosis factor receptor 2; FACS: Fluorescence-activated cell sorting.

## Supplementary Tables

**Supplementary Table 1.** Drug developability of Nanobody-161 antibody.

| Parameter | SDS-PAGE purity | SEC | pI | Tm | Yield  (μg/mL) | Polyspecificity | Immunogenicity risk |
| --- | --- | --- | --- | --- | --- | --- | --- |
| Nanobody-161 | 96.69 | 100 | 7.95 | 65.68 | 2030 | N/A | Low |

**Supplementary Table 2.** Key interactions in TNFR2-Nanobody-161 complex.

|  | No | Nanobody-161 | Distance [Å] | TNFR2 |
| --- | --- | --- | --- | --- |
| Salt bonds | 1 | R29-NH1 | 3.30 | E110-OE2 |
|  | 2 | R29-NH2 | 3.81 | E110-OE1 |
| Hydrophobic interactions | 1 | F30-CE2 | 4.99 | I95-CG2 |
|  | 2 | F107-CE2 | 3.27 | V83-CG1 |
|  | 3 | F107-CD2 | 4.92 | P100-CD |
| Hydrogen bonds | 1 | R53-NE | 3.40 | E84-O |
|  | 2 | S101-OG | 3.17 | E133-OE1 |
|  | 3 | Q102-N | 3.11 | E133-OE2 |
|  | 4 | L103-N | 3.56 | E133-OE1 |
|  | 5 | G104-N | 3.74 | E133-OE1 |
|  | 6 | Y105-N | 3.65 | E133-OE1 |
|  | 7 | Y105-OH | 3.76 | K108-O |
|  | 8 | A106-N | 3.29 | E133-OE1 |
|  | 9 | F107-N | 2.88 | C98-O |
|  | 10 | R108-NH2 | 3.74 | G131-O |

Abbreviation: TNFR2: Tumor necrosis factor receptor 2.

**Supplementary Table 3.** Binding affinity constants (K_D_) of Nanobody-161 WT and mutants interacting with TNFR2-His.

| No. | Nanobody-161 | KD (M) | Ka (1/Ms) | Kd (1/s) | R^2^ |
| --- | --- | --- | --- | --- | --- |
| 1 | WT | 8.15E-08 | 3.39E+03 | 2.76E-04 | 0.996 |
| 2 | R29A | 6.31E-07 | 3.17E+03 | 2.00E-03 | 0.997 |
| 3 | F30G | 3.78E-06 | 3.34E+03 | 1.26E-02 | 0.984 |
| 4 | R53A | 7.95E-08 | 4.03E+03 | 3.21E-04 | 0.993 |
| 5 | S101A | 1.72E-06 | 5.37E+03 | 9.26E-03 | 0.986 |
| 6 | Q102A | 2.12E-07 | 3.29E+03 | 6.99E-04 | 0.996 |
| 7 | L103G | 9.76E-07 | 3.92E+03 | 3.82E-03 | 0.996 |
| 8 | Y105G | 2.14E-04 | 4.29E+02 | 9.17E-02 | 0.963 |
| 9 | F107G | N/A | N/A | N/A | N/A |
| 10 | R108A | 1.86E-07 | 4.73E+03 | 8.77E-04 | 0.994 |

Abbreviations: TNFR2: Tumor necrosis factor receptor 2; WT: wild type.

**Supplementary Table 4.** Survival rates in the MC38 tumor-bearing humanized TNFR2 mouse model.

| Treatment | Percent survival (%) | | | | | |
| --- | --- | --- | --- | --- | --- | --- |
|  | D17 (N) | D21 (N) | D24 (N) | D28 (N) | D31 (N) | D35 (N) |
| Nanobody-161, 7.5 mpk | 100 (6/6) | 100 (6/6) | 83 (5/6) | 67 (4/6) | 67 (4/6) | 67 (4/6) |
| Nanobody-161, 2.5 mpk | 100 (6/6) | 100 (6/6) | 100 (6/6) | 100 (6/6) | 100 (6/6) | 100 (6/6) |
| Nanobody-161, 0.5 mpk | 100 (6/6) | 100 (6/6) | 100 (6/6) | 100 (6/6) | 100 (6/6) | 100 (6/6) |
| OPI-SBT002e, 14.42 mpk | 100 (6/6) | 100 (6/6) | 100 (6/6) | 100 (6/6) | 100 (6/6) | 100 (6/6) |
| OPI-SBT002e, 4.81 mpk | 100 (6/6) | 100 (6/6) | 100 (6/6) | 100 (6/6) | 100 (6/6) | 100 (6/6) |
| OPI-SBT002e, 0.96 mpk | 100 (6/6) | 100 (6/6) | 100 (6/6) | 100 (6/6) | 100 (6/6) | 100 (6/6) |
| PBS | 100 (8/8) | 100 (8/8) | 100 (8/8) | 100 (8/8) | 100 (8/8) | 100 (8/8) |

Abbreviations: TNFR2: Tumor necrosis factor receptor 2.
